# Draft genome sequence of *Venturia carpophila*, the causal agent of peach scab

**DOI:** 10.1186/s40793-017-0280-0

**Published:** 2017-12-02

**Authors:** Chunxian Chen, Clive H. Bock, Bruce W. Wood

**Affiliations:** USDA, Agricultural Research Service, Southeastern Fruit and Tree Nut Research Lab, 21 Dunbar Road, Byron, GA 31008 USA

**Keywords:** *Cladosporium carpophilum*, *Fusicladosporium carpophilum*, *Prunus persica*, Fungal pathogen, Venturiacae

## Abstract

*Venturia carpophila* causes peach scab, a disease that renders peach (*Prunus persica*) fruit unmarketable. We report a high-quality draft genome sequence (36.9 Mb) of *V. carpophila* from an isolate collected from a peach tree in central Georgia in the United States. The genome annotation is described and a phylogenetic analysis of the pathogen is presented. The genome sequence will be a useful resource for various studies on the pathogen, including the biology and ecology, taxonomy and phylogeny, host interaction and coevolution, isolation and characterization of genes of interest, and development of molecular markers for genotyping and mapping.

## Introduction

**Fig. 1:**
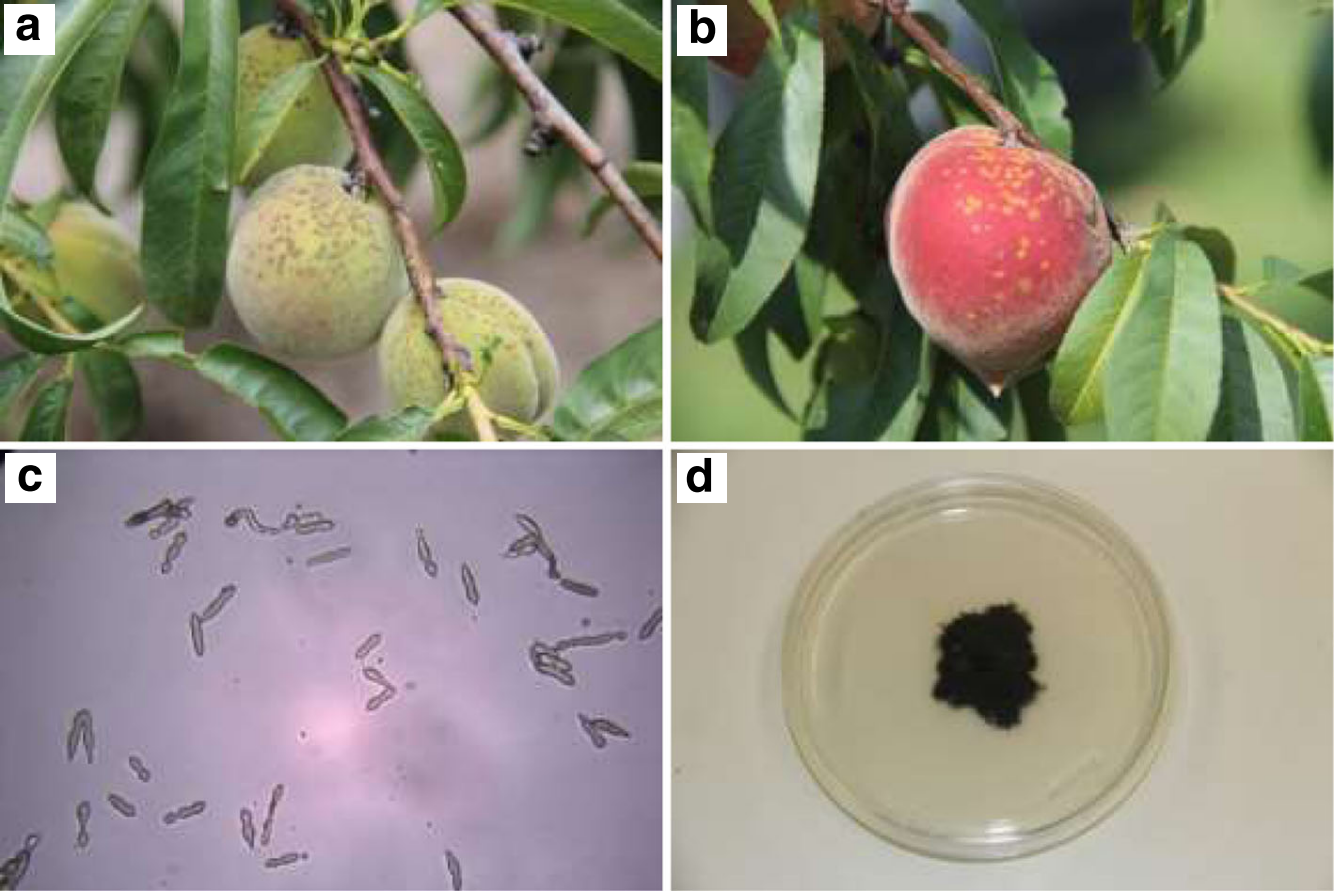
Scab symptoms caused by *Venturia carpophila* on **a** immature peach fruit, **b** a ripe peach fruit, **c** conidia of *V. carpophila* (400×), and **d** an 8-week old colony of *V. carpophila* growing on potato dextrose agar

*Venturia carpophila* E.E. Fisher (syn. *Fusicladosporium carpophilum* Partridge and Morgan Jones; *Cladosporium* *carpophilum* Thüm.; *Megacladosporium carpophilum* Thüm.(Vienn.-Bourg); *Fusicladium* *carpophilum* Thüm (Oudem.); *Cladosporium* *americanum* H.C. Greene) is an important fungal pathogen in the family Venturiaceae and is the causal agent of scab in peach (*Prunus persica*) and other species of *Prunus* [1]. Typical symptoms on fruit are black freckles, spots, and/or scabs of variable size, distribution and density (Fig. 1 a and b) that render the fruit unfit for market [2]. The pathogen also infects shoots and overwinters in lesions on 1-year-old twigs, which are thought to be the source of primary inoculum in the form of conidia that are both airborne and splashborne, resulting in infection of young fruit during the spring and early summer [3]. Peach scab generally develops on the most exposed, easily wetted, uppermost portion of the fruit surface, which is consistent with the splash-borne nature of conidia [4]. Multiple fungicide sprays are required to control peach scab at variable costs, which impose a risk of the pathogen developing resistance to certain fungicides. The effect of the fungicide sprays in reducing disease severity and thus improving fruit quality has been demonstrated, but under certain conditions the results are inconsistent [5].

Besides peach, several economically important stone fruit crops, including apricot (*P. armeniaca*), almond (*P. dulcis*), and plum (*P. domestica*), can be infected by *V. carpophila* [1]. Only recently has the taxonomic identity of the pathogens causing scab on stone fruit and other related genera begun to be clarified [6–8]. No complete genome sequence of *V. carpophila* has been reported, although some related species have now been sequenced [9–11]. As with these other species, an annotated genome of *V. carpophila* is a valuable resource for various genomic, genetic, and systematic studies. For example, various genes of interest and importance, such as those related to fungicide resistance, host recognition, or mating type, can be identified for further research to aid in management of the disease. Microsatellites can be developed as informative markers for genetic mapping and diversity studies. Also, the knowledge obtained from the genome can be useful in improving development of resistant cultivars.

In this report, we describe the first high-quality draft genome sequence of *V. carpophila* and provide a phylogenetic analysis of the fungus and other closely related species. The genome sequence will facilitate further genomic and phylogenetic exploration to understand the pathogen and its relationship with peach.

## Organism information

### Classification and features

The sequenced strain of *V. carpophila* was isolated from a scab-infected peach fruit (cv. Julyprince) from an 8-year-old tree in June 2014 at the USDA-ARS-SEFTNRL, Byron, Georgia, USA (Table 1). Asexually produced conidia (Fig. 1c) of *V. carpophila* were scraped from a single lesion on the fruit surface using a scalpel, and a dilute spore solution prepared in sterile distilled water. Multiple 0.1 μL aliquots were spread on the surface of replicate water agar plates, amended with lactic acid (0.50 mL/L), streptomycin (0.20 g/L), tetracycline (0.05 g/L) and chloramphenicol (0.05 g/L). Plates were incubated at 27 °C for 48 h under fluorescent light on a 12/12 h day/night cycle. A single germinated spore of *V. carpophila* was excised on an agar plug using a scalpel under a microscope (50×), and was transferred to antibiotic-amended potato dextrose agar, and was grown for 4 weeks under the same conditions described above (Fig. 1d).

**Table 1:** Classification and general features of *Venturia carpophila*

MIGS ID	Property	Term	Evidence code ^a^
	Classification	Domain *Fungi*	TAS [35]
		Phylum *Ascomycota*	TAS [35]
		Class *Dothidiomycetes*	TAS [36]
		Order *Pleosporales*	TAS [37]
		Family *Venturiaceae*	TAS [37]
		Genus *Venturia*	TAS [38]
		Species *Venturia carpophila*	TAS [1]
		Strain JB3–5	NAS
	Gram stain	N/A	NAS
	Cell shape	Not reported	NAS
	Motility	Not reported	NAS
	Sporulation	Conidia and ascospores	TAS [1, 2]
	Temperature range	Mesophilic (15–25 °C)	TAS [39]
	Optimum temperature	Not reported	NAS
	pH range; Optimum	Not reported	NAS
	Carbon source	Not reported	NAS
	Dispersal	Rain splash and wind	TAS [3, 39]
	Infection	Surface wetness	TAS [3]
MIGS-6	Habitat	Arboreal	TAS [2]
MIGS-6.3	Salinity	Not reported	NAS
MIGS-22	Oxygen requirement	Aerobic	NAS
MIGS-15	Biotic relationship	Parasite	TAS [2]
MIGS-14	Pathogenicity	Pathogenic	TAS [2]
MIGS-4	Geographic location	Byron, Georgia, USA	NAS
MIGS-5	Time of sample collection	July 2010	NAS
MIGS-4.1	Latitude	32.652^o^ N	NAS
MIGS-4.2	Longitude	83.739 ^o^ W	NAS
MIGS-4.4	Altitude	156 m	NAS

^a^ Evidence codes – IDA: Inferred from Direct Assay; TAS: Traceable Author Statement (i.e., a direct report exists in the literature); NAS: Non-traceable Author Statement (i.e., not directly observed for the living, isolated sample, but based on a generally accepted property for the species, or anecdotal evidence). Evidence codes as for the Gene Ontology project [40]

The fungus belongs in the Eukaryota, is a member of the Fungal kingdom, phylum Ascomycota, class Dothidiomycetes, and family Venturiaceae (Table 1). Several other economically important plant pathogens are members of the Dothidiomycetes, including apple scab (*V. inaequalis*), pear scab (*V. pyrina*), pecan scab (*F. effusum*), rice scald (*Magnaprthe oryzae*), and Septoria leaf blotch of wheat (*Zymoseptoria tritici* syn. *Mycosphaerella* *graminicola*). *V. carpophila* has been classified based on its host range, morphology and some molecular characteristics [1]. The sexual stage (pseudothecia that produce ascospores) of the fungus has been identified and described from Australia [1], but has not been described elsewhere at any time. Its role in the epidemiology of the disease is unknown.

**Fig. 2:**
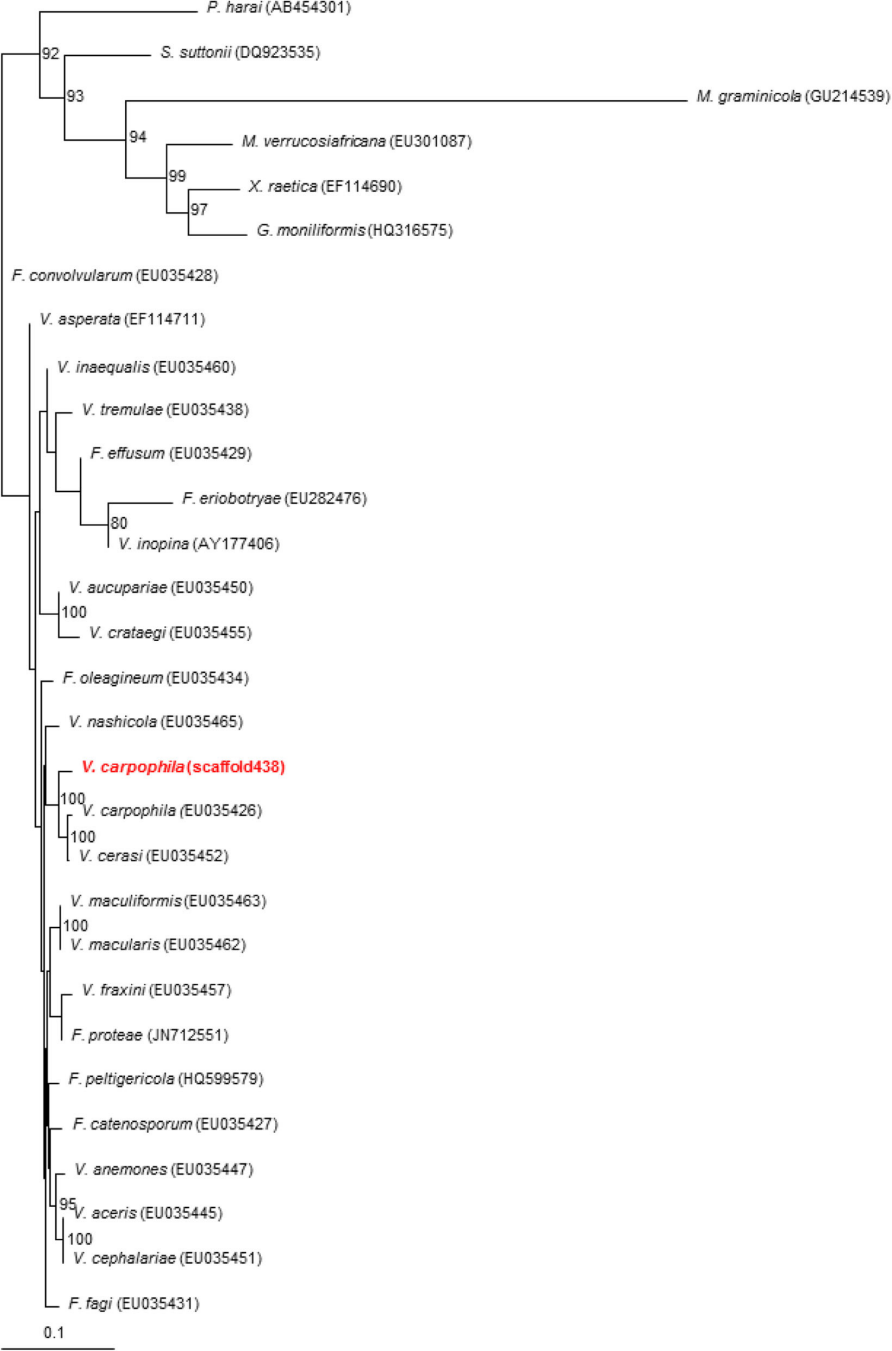
The phylogenetic position of *Venturia carpophila* in comparison with other related fungal species. The tree was developed based on the 18S rRNA gene of the sequenced isolate of *V. carpophila*, an accession of the 18S rRNA gene of another isolate of *V. carpophila*, and accessions of other closely-related members of the family Venturiaceae (genera *Fusicladium* and *Venturia*) and with outgroup representatives (*Phyllosticta harai*, *Staniwardia suttonii*, *Mycosphaerella graminicola*, *M. verrucosiafricana*, *Xenomeris raetica* and *Gibberella moniliformis*, all in the class Dothidiomyectes of the phylum Ascomycota). The nearest neighbor joining tree was built by CLUSTALX2 [12] and drawn to scale by TreeView [13] based on 18S rRNA sequence phylogenetic analysis involved in a 1000-replicate bootstrapping process (numbers adjacent to branches are support values from 1000 bootstraps). The branch lengths in the tree were measured as substitutions per site, i.e., 0.1 on the scale bar representing 4 substitutions in 100 bp. The evolutionary history was inferred from 1522 aligned characters. The GenBank accession numbers for each species or strain are shown in parenthesis

The phylogenetic relationship of *V. carpophila* to other Ascomycota species based on the 18S rRNA genes shows that it is most closely related to members of the family Venturiaceae, particularly the genera *Fusicladium* and *Venturia* (Fig. 2). The 18S rRNA gene was located on scaffold_438 and a 1552 bp sequence aligned with the sequences from the other fungi was used for the analysis. The phylogenetic analysis was performed using the nearest neighbor joining method in CLUSTALX2 [12] with node values based on 1000 replicates. The phylogenetic tree was drawn by TreeView [13]. Members from other genera in the Dothidiomycetes were included as outgroups.

## Genome sequencing information

### Genome project history

The genome of *V. carpophila* described here was sequenced in 2015 at the ICBR core facility of the University of Florida, Gainesville, Florida, USA. The genome was assembled and annotated at the USDA-ARS Fruit and Tree Nut Research Laboratory. This Whole Genome Shotgun project has been deposited at DDBJ/ENA/GenBank under the accession MECS00000000 (Bioproject PRJNA321389). The version described in this paper is version MECS01000000. The project data is summarized in Table 2. The project information is in compliance with MIGS version 2.0 [14].

**Table 2:** Project information

MIGS ID	Property	Term
MIGS-31	Finishing quality	High-quality draft
MIGS-28	Libraries used	A paired-end library (average insert 518 bp for 2 × 300 cycles)
MIGS-29	Sequencing platforms	Illumina Miseq
MIGS-31.2	Fold coverage	263×
MIGS-30	Assembler	A5-MiSeq
MIGS-32	Gene calling method	Augustus using *Saccharomyces* as the species parameter, also COG and BLAST search NCBI NR (non-redundant) database
	Locus Tag	N/A
	Genbank ID	MECS00000000
	Genbank Date of Release	2017–02-10
	GOLD ID	N/A
	BIOPROJECT	PRJNA321389
	BIOSAMPLE	SAMN04993191
MIGS-13	Source Material Identifier	N/A
	Project relevance	Biotechnology/mycology/disease control

### Growth conditions and genomic DNA preparation

Culture of *V. carpophila* was on antibiotic-amended potato dextrose agar. The culture was incubated for 4 weeks at 25 °C (12 h light/12 h dark), when the DNA was extracted from the sample using a ZymoResearch DNA extraction kit (ZymoResearch, Irvine, CA), following a slightly modified protocol for DNA extraction from fungi [15]. A Qiagen Tissue Lyser (Qiagen, Valencia, CA) was used to lyse the mycelium. Once obtained, the DNA was quantified using a Nanodrop spectrophotometer (Nanodrop Products, Wilmington, DE) and stored in TE buffer at −20 °C.

### Genome sequencing and assembly

The genome was sequenced using an Illumina paired-end library (a V3 kit, 2 × 300 cycles) and a MiSeq machine, which generated 400,041,052 raw reads consisting of 12,052,356,652 raw nucleotides. The A5-Miseq assembly pipeline was used automatically to check quality, trim adaptors, filter low-quality reads, to correct sequencing errors using robust error correction (EC) parameters, and generate high-quality genome contigs with additional detection of assembly errors [16]. About 97.54% raw reads and 83.90% nucleotides passed EC; thus a total of 39,057,608 EC reads, containing 10,111,608,273 EC nucleotides, were subject to the final assembly process. A total of 657 contigs, accounting for 36,917,822 bp, were assembled, representing the assembled genome size of the pathogen. Of the assembled nucleotides, 98.58% bases had a PHRED-scale score quality > = 40 (Q40) and the average depth of each nucleotide was 263.47, indicating it is a high-quality assembly. Additionally, the longest contig is 1,454,817 bp and the N50 length is 292,586 bp, suggesting the genome was covered mostly by larger contigs. The actual genome size is unknown at this stage, but the 263 × genome coverage likely covers more than 95% of the genome. Therefore we can estimate that the genome size of *V. carpophila* is ~38.9 Mb, which is in the typical size range of genomes in the phylum Ascomycota [17].

### Genome annotation

The draft genome was annotated using the MAKER pipeline [18]. In summary, repeats were first found and masked using RepeatMasker and the RepBase database [19]; ab initio gene prediction was performed with AUGUSTUS under the parameter *Saccharomyces* [20]; these predicted genes were annotated by BLAST against the NCBI non-redundant (nr) nucleotide database and also by RPSBLAST (Reverse Position-Specific BLAST) batch search in conserved domain database (CDD v3.14) [21, 22]. The CCD is a superset including a total of 47,363 position-specific scoring matrix (PSSM) domains curated in the NCBI and imported from Pfam [23], SMART [24], COG [25], PRK [26], and TIGRFAM [27]. The e-value for BLAST and RPSBLAST search in a database was 1e-50 and 0.01, respectively. In addition, CRISPR regions were identified using the CRISPR Recognition Tool (CRT) [28]; tRNAs were identified by tRNAScan-SE-1.23 [29]; rRNAs were identified by RNAmmer [30]; signal peptides and transmembrane helices were predicted using SignalP [31] and TMHMM [32], respectively. According to BLASTN, 107 of the 657 contigs, accounting for 144,247 bp, only had multiple hits of mitochondrial genome sequences at e-10, suggesting they belong to the organelle genome of the pathogen.

## Genome properties

The properties of the *V. carpophila* genome are summarized in Table 3. The draft genome sequence had 36,917,822 bp in 657 contigs, with a G + C content of 47.36%. The G + C content of the 550 contigs presumable for the nuclear genome is 47.43% and that of the 107 contigs likely belonging to the mitochondrial genome is 30.13%. Of the total 8220 predicted protein-coding genes, 6547 had hits in the nr database. In addition, 4632 had predicted functions, 2694 had Pfam domains, and 3665 were assigned to COGs. The distribution of genes into COG functional categories is presented in Table 4.

**Table 3:** Nucleotide and gene count levels of the genome

Attribute	Value	% of Total ^a^
Genome size (bp)	36,917,822	
DNA coding (bp)	10,768,752	29.17
DNA G + C (bp)	17,485,709	47.36
DNA scaffolds	657	
Total genes	8352	
Protein coding genes	8220	98.42
RNA genes	132	1.58
Pseudo genes	Not reported	
Genes in internal clusters	Not reported	
Genes with function prediction	4632	56.35
Genes assigned to COGs	1136	13.82
Genes with Pfam domains	2694	32.77
Genes with signal peptides	390	4.74
Genes with transmembrane helices	2622	31.90
CRISPR repeats	4	

^a^ The total is based on either the size of the genome in base pairs or the total number of protein coding genes in the annotated genome

**Table 4:** Number of genes associated with the 25 general COG functional categories

Code	Value	% of total ^a^	Description
J	200	2.43	Translation
A	21	0.26	RNA processing and modification
K	136	1.65	Transcription
L	208	2.53	Replication, recombination and repair
B	28	0.34	Chromatin structure and dynamics
D	172	2.09	Cell cycle control, mitosis and meiosis
Y	4	0.05	Nuclear structure
V	63	0.77	Defense mechanisms
T	122	1.48	Signal transduction mechanisms
M	142	1.73	Cell wall/membrane biogenesis
N	19	0.23	Cell motility
Z	24	0.29	Cytoskeleton
W	–	–	Extracellular structures
U	70	0.85	Intracellular trafficking and secretion
O	295	3.59	Posttranslational modification, protein turnover, chaperones
C	240	2.92	Energy production and conversion
G	249	3.03	Carbohydrate transport and metabolism
E	270	3.28	Amino acid transport and metabolism
F	58	0.71	Nucleotide transport and metabolism
H	134	1.63	Coenzyme transport and metabolism
I	217	2.64	Lipid transport and metabolism
P	144	1.75	Inorganic ion transport and metabolism
Q	117	1.42	Secondary metabolites biosynthesis, transport and catabolism
R	622	7.57	General function prediction only
S	110	1.34	Function unknown
–	4555	55.41	Not in COGs

^a^ The total is based on the total number of protein coding genes in the annotated genome

## Insights from the genome sequence

The genome provides a useful resource for identifying genes of interest in *V. carpophila*. Furthermore, the phylogenetic analysis presented earlier confirms the relationship of *V. carpophila* to other members of the Venturiacae and confirms previous observations on the taxonomic relationships among these members of the Ascomycota. Based on the phylogenetic analysis using the sequence of the 18S rRNA gene (Fig. 2), *V. carpophila* is closely related to other scab-causing fungal pathogens of higher plants, including *V. cerasi*, causing scab on cherry, and also *V. nashicola,* cause of scab on Asian pear.

### Extended insights

**Fig. 3:**
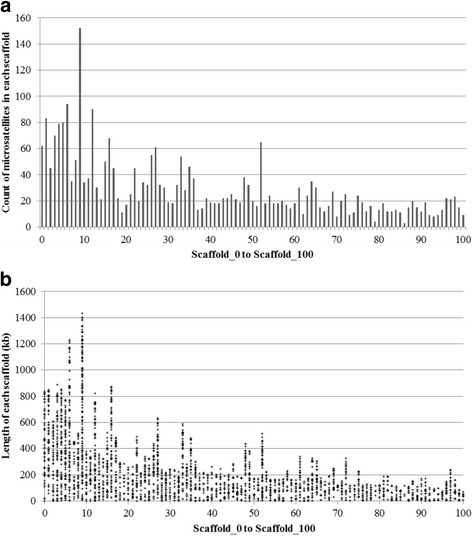
Count (**a**) and distribution (**b**) of microsatellites on scaffold_0 to scaffold_100. The total length of the 101 scaffolds (0 to 100) is 31,436,433 bp, accounting for 85.15% of the total genome size and containing 80.10% of the microsatellites. Scaffold_9 is the longest (1,454,817 bp) and has the most microsatellites (152). In b, each cross mark represents a microsatellite at the corresponding position on that scaffold

The genome of the peach scab fungus, *V. carpophila*, will be of great value to research on this organism moving forward. Apart from the opportunity it affords for more detailed analysis of individual genes relating to the secretome, we have identified several genes of interest and all microsatellites for further characterization. First, we have located the full open reading frame for one of the mating type idiomorph genes (*MAT1–2*) in scaffold_15 (between 199,706–200,916 bp) with two predicted introns (between 199,953–200,006 bp and between 200,318–200,365 bp), which paves the way for a better understanding of the reproduction of this fungus, particularly in the USA [1]. When fully characterized, the mating type idiomorphs provide the basis not only for a more complete understanding of the epidemiology of the pathogen, but also the potential opportunity for management of the disease through control of the sexual stage, as has been demonstrated with the closely related apple scab pathogen [33]. Secondly, we have also identified the cytochrome b gene in scaffold_347 (between 2149 and 7821 bp), which contains at least two introns. The accurate boundaries of the introns need further determination by PCR and sequencing of cDNA. This gene is particularly important in plant pathogenic fungi as it plays a critical role in resistance to the quinone outside inhibitor fungicides (also known as strobilurins). Understanding the structure of this gene will form the basis for assessing risk of resistance to the quinone outside inhibitors, as demonstrated in closely related pathogens [34]. Thirdly, we have identified a total of 4021 microsatellites in 322 of the 657 scaffolds. On average, there was about one microsatellite per 10 kb of genome. The tri-type microsatellite was the most abundant. The total length of the largest 101 scaffolds (0 to 100) is 31,436,433 bp, accounting for 85.15% of the total genome size and containing 80.10% of the microsatellites. The count and distribution of microsatellites on scaffold_0 to scaffold-100 are presented (Fig. 3 a and b). Scaffold 9 had the highest count of microsatellites (152), and it was also the longest scaffold. Microsatellites are basic to studying the genetic diversity and population structure of the pathogen, and also have potential to be used for tracking specific genes of interest.

## Conclusions

The predicted genes may represent most functional genes in the *V. carpophila* genome and can be used as a new resource for developing molecular markers for genetic diversity studies, and for other research into the biology, ecology, taxonomy and phylogeny of the pathogen, and for research into host/pathogen coevolution.
